# SARS-CoV-2 Receptor-Binding Domain IgG Response to AstraZeneca (AZD1222) COVID-19 Vaccination, Jamaica

**DOI:** 10.4269/ajtmh.21-1128

**Published:** 2022-03-23

**Authors:** Ynolde E. Leys, Magdalene Nwokocha, Jerome P. Walker, Tiffany R. Butterfield, Velesha D. Frater, Tamara K. Thompson, Mark Anderson, Gavin A. Cloherty, Joshua J. Anzinger

**Affiliations:** ^1^ Department of Microbiology, The University of the West Indies, Kingston, Jamaica, West Indies;; ^2^ Department of Pathology, The University of the West Indies, Kingston, Jamaica, West Indies;; ^3^ Department of Medicine, The University of the West Indies, Kingston, Jamaica, West Indies;; ^4^ Infectious Disease Research, Abbott, Abbott Park, Illinois;; ^5^ Global Virus Network, Baltimore, Maryland

## Abstract

The Caribbean region is lacking an assessment of the antibody response and side effects experienced after AstraZeneca COVID-19 vaccination (AZD1222). We examined severe acute respiratory syndrome coronavirus 2 (SARS-CoV-2) spike receptor-binding domain (RBD) IgG levels and report the side effects noted in a Jamaican population after AZD1222 vaccination. Median RBD IgG levels for persons without evidence of previous SARS-CoV-2 infection were 43.1 binding international units (bIU)/mL 3 to 7 weeks after the first dose, increasing to 100.1 bIU/mL 3 to 7 weeks after the second dose, and decreasing to 46.9 bIU/mL 16 to 22 weeks after the second dose. The median RBD IgG level 2 to 8 weeks after symptom onset for unvaccinated SARS-CoV-2-infected persons of all disease severities was 411.6 bIU/mL. Common AZD1222 side effects after the first dose were injection site pain, headache, and chills. Most people reported no side effects after the second dose. AZD1222 is widely used across the English-speaking Caribbean, and our study provides evidence for its continued safe and effective use in vaccination programs.

## INTRODUCTION

The antibody response to COVID-19 vaccination is considered to be of critical importance for protection from COVID-19, especially severe manifestations.^1^ In general, antibody responses to vaccination can differ depending on the population examined,^2^ and ideally should be assessed for each population. All Caribbean Community member states have received AstraZeneca vaccines (AZD1222) through COVAX and/or donations, representing the majority of all vaccine types.^3^ Thus far, there have been no studies examining the antibody response to AZD1222 in the Caribbean, necessitating public health decisions based on data from different populations that may use different intervals between vaccine doses.

Jamaica was the first Caribbean country to receive AstraZeneca COVID-19 vaccines via COVAX, with the first administered on March 10, 2021, exactly 1 year after the first confirmed case in the country.^4^ In our study, we examined the antibody response and side effects experienced after AZD1222 vaccination for a group of initial vaccinees at the University of the West Indies and the University Hospital of the West Indies, Jamaica, and compared responses to unvaccinated severe acute respiratory syndrome coronavirus 2 (SARS-CoV-2)-infected persons. Participants were primarily health-care workers and faculty, but also included other persons associated with both institutions. Antibody responses were assessed for people receiving AZD1222 first doses from March 10 through April 27, 2021. Second-dose appointments in Jamaica were made available 2 calendar months after the first vaccination, with the full-dose AZD1222 (5 × 10^10^ viral particles) offered exclusively for these months. This study was approved by the University of the West Indies Mona Campus Research Ethics Committee (CREC-MN.150 20/21).

Antibody responses to vaccination were determined using an Abbott ARCHITECT *i*2000sr instrument (Abbott Laboratories, Abbott Park, IL) for the SARS-CoV-2 IgG II Quant and Abbott ARCHITECT SARS-CoV-2 IgM assays. Both identified antibodies specific for the spike protein. The SARS-CoV-2 IgG II Quant assay measured antibodies against the spike receptor-binding domain (RBD), the domain responsible for binding to angiotensin-converting enzyme 2 receptors and a major target of neutralizing antibodies.^5^ SARS-CoV-2 IgG II Quant assay results are reported in WHO binding international units (bIU) per milliliter by multiplying the arbitrary units (AUs)-per-milliliter value by 0.142.^6^ Because spike RBD IgG units can differ between assays, the use of bIUs per milliliter allows for an international standard unit. Past infection with SARS-CoV-2 was determined using the Abbott ARCHITECT SARS-CoV-2 IgG assay that identified nucleocapsid-specific IgG. A lower cutoff (≥ 0.4 signal-to-cutoff [S/CO]) than the manufacturer’s instructions was used for the SARS-CoV-2 nucleocapsid-specific IgG assay to increase sensitivity, as described elsewhere.^7^ All samples in our study were tested with all three assays. SARS-CoV-2 IgG II Quant and IgM assays were considered positive according to the manufacturer’s instructions (≥ 50 AU/mL and ≥ 1.0 S/CO, respectively).

**Figure 1.**
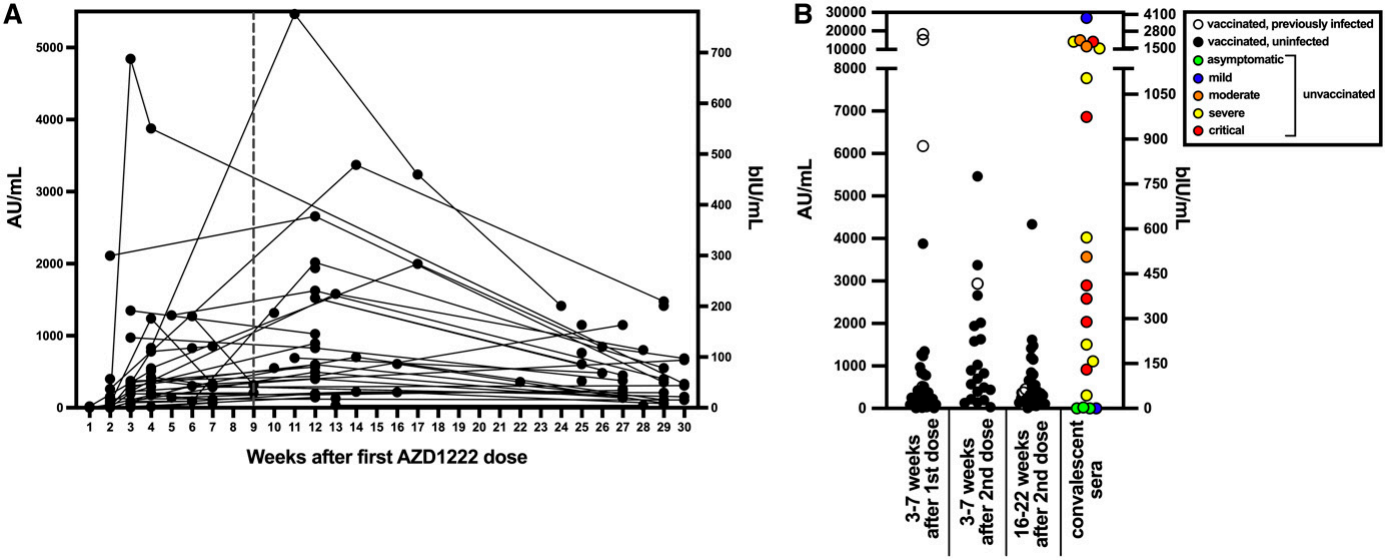
Severe acute respiratory syndrome coronavirus disease 2 (SARS-CoV-2) receptor-binding domain (RBD) IgG antibody levels after AZD1222 vaccination and/or SARS-CoV-2 infection. SARS-CoV-2 RBD IgG was measured with the Abbott ARCHITECT SARS-CoV-2 IgG II Quant assay with results reported as arbitrary units (AU) per milliliter on the left *y*-axis and WHO binding international units (bIU) per milliliter on the right *y*-axis. (**A**) Sera from all time points collected after AZD1222 vaccination were assessed for SARS-CoV-2 RBD IgG levels. The vertical dotted line indicates the average time of the second AZD1222 dose. All data points to the right of the vertical dotted were after the second AZD1222 dose. (**B**) Sera were collected from AZD1222-vaccinated persons 3 to 7 weeks after the first and second doses, 16 to 22 weeks after the second dose, and for unvaccinated SARS-CoV-2 polymerase chain reaction (PCR)-confirmed persons 2 to 8 weeks after symptom onset (or PCR confirmation for asymptomatic persons).

A total of 71 AZD1222-vaccinated people were assessed; 52.1% were male and the study population had an average age of 49.9 years (Table 1). The average time between doses was 9.0 ± 0.8 weeks. Three weeks after first dose, all but one person was positive for SARS-CoV-2 RBD IgG, and most people with longitudinal samples showed an increased RBD IgG level after the second AZD1222 dose that decreased over time (Figure 1A). In participants without serological evidence of previous SARS-CoV-2 infection, median RBD IgG responses were 43.1 bIU/mL (303.5 AU/mL) 3 to 7 weeks after the first dose, increasing to 100.1 bIU/mL (704.6 AU/mL) 3 to 7 weeks after the second dose, and decreasing to 46.9 bIU/mL (330.3 AU/mL) 16 to 22 weeks after the second dose (Figure 1B and Table 1). The only person not showing evidence of RBD IgG antibodies (< 50 AU/mL) after the second AZD1222 dose was notable for an age of more than 80 years (the only participant older than 80 years).

**Table 1:** Participant demographic information and severe acute respiratory syndrome coronavirus disease 2 receptor-binding domain IgG levels

Characteristic	Vaccinated				Infected, unvaccinated; 2 to 8 weeks after symptom onset*
	Three to 7 weeks after first AZD1222 dose	Three to 7 weeks after second AZD1222 dose	Sixteen to 22 weeks after second AZD1222 Dose	All time points after AZD1222	
No. of patients	39	21	36	71	21
Male	66.7%	76.2%	66.7%	52.1%	52.4%
Mean age ± SD	50.4 ± 16.1 y	54.8 ± 14.5 y	51.4 ± 16.6 y	49.1 ± 15.5 y	54.1 ± 17.4 y
SARS-CoV-2 RBD IgG, AU/mL (IQR); all samples	305.8 (91.8–792.9)	827.0 (310.5–1,978.9)	342.0 (168.2–681.0)	N/A	2,898.9 (611.1–11,141.1)
SARS-CoV-2 RBD IgG, AU/mL (IQR); excluding previous infection	303.5 (91.8–606.2)	704.6 (310.5–1,978.9)	330.3 (146.2–607.1)	N/A	N/A

AU = arbitrary units; IQR = interquartile range; N/A = not applicable; RBD = receptor-binding domain; SARS-CoV-2 = severe acute respiratory syndrome coronavirus disease 2.

* Two to eight weeks after positive severe acute respiratory syndrome coronavirus disease 2 polymerase chain reaction test for asymptomatic infections.

Similar to previous studies with AZD1222 and Pfizer-BioNTech COVID-19 (BNT162b2) vaccines,^8^^,^^9^ most vaccinated persons with evidence of previous SARS-CoV-2 infection showed greater RBD IgG levels after the first dose than people vaccinated without evidence of previous SARS-CoV-2 infection (Supplemental Figure S1). The three persons with the greatest RBD IgG levels after AZD1222 vaccination had SARS-CoV-2 polymerase chain reaction (PCR)-confirmed infection within 2 months before receiving the first dose (Supplemental Table S1). In total, 12 people showed serological evidence of SARS-CoV-2 infection, with four persons experiencing COVID-19 (three SARS-CoV-2 PCR-confirmed and one previously antibody positive) and eight not reporting COVID-19. One person showed serological evidence of SARS-CoV-2 infection after the first but before the second AZD1222 dose, and three persons showed evidence of a SARS-CoV-2 breakthrough infection 3 to 18 weeks after the second dose of AZD1222 (Supplemental Figure S2). The three people with serological evidence of infection after the second dose of AZD1222 had spike RBD IgG values of 20.4 bIU/mL (143.8 AU/mL), 126.8 bIU/mL (893.2 AU/mL), and 145. bIU/mL (1,024.7 AU/mL). These data indicate that after two doses of AZD1222, even with a spike RBD IgG value of 145.5 bIU/mL (1,024.7 AU/mL)—a value greater than the median response—breakthrough infection may still occur. Sterilizing immunity in the respiratory tract is not expected with COVID-19 vaccines given intramuscularly,^10^ but may still provide protection from disease. Notably, none of the persons in our study with serological evidence of breakthrough infection reported COVID-19 symptoms. Positive SARS-CoV-2 IgM results were generally not observed after AZD1222 vaccination (Supplemental Figure S3), and index values were much lower than we reported previously for SARS-CoV-2 natural infection.^11^

For comparison to AZD1222 vaccination, the RBD IgG response was assessed with convalescent sera from 21 unvaccinated SARS-CoV-2 PCR-confirmed persons (52.4% male; average age, 54.1 years) (Table 1). RBD IgG responses were assessed 2 to 8 weeks after symptoms onset (or positive PCR test for asymptomatic infections) for unvaccinated persons infected during the initial (April 2020–January 2021) and third (July–September 2021) waves (Figure 1B and Table 1). Both waves were grouped together because median RBD IgG responses were not statistically different (Mann-Whitney test, *P* > 0.999) when comparing severe–critical infections for the two waves (only severe–critical infection data were available for the third wave). The median RBD IgG antibody response for unvaccinated PCR-confirmed SARS-CoV-2-infected people was 411.6 bIU/mL (2,898.9 AU/mL) when including all WHO disease severities. Greater RBD IgG antibody levels correlated with disease severity as assessed by Spearman’s correlation (ρ = 0.44, *P* = 0.04), in agreement with previous observations.^12^ No correlation was identified for RBD IgG levels and age or gender for unvaccinated SARS-CoV-2-infected persons as assessed by Spearman’s correlation. The availability of convalescent sera from SARS-CoV-2 people was limited for our study and does not represent the general population, in which most infections have been described as asymptomatic and mild.^13^ Thus, direct comparison of median antibody responses between vaccinees and convalescent persons in our study is not appropriate, and comparisons of antibody responses between vaccinees and convalescent patients of differing disease severity should be interpreted cautiously as a result of the limited number of convalescent samples available for our study.

Most people reported side effects and treatment after the first AZD1222 dose, whereas after the second dose, fewer people reported side effects; if they did, the effects were of shorter duration (Table 2). Spearman’s correlation indicated that myalgia, arthralgia, and eye pain each correlated individually with RBD IgG levels (ρ = 0.385, *P* = 0.02; ρ = 0.374, *P* = 0.03, ρ = 0.368, *P* = 0.03, respectively) after the first dose, but no correlation between any side effect and RBD IgG level was identified after the second dose. Side effects after the first dose were mostly similar to those reported for a phase 1/2 safety and immunogenicity study in a UK population, with the exception of lower percentages reporting fatigue (18% versus 70–71%) and myalgia (16% versus 48–60%) in our study compared with the UK study.^14^

**Table 2:** Side effects and treatments after AZD1222 vaccination

Side effects	First dose	Second dose
None	19.6% (11/56)	55.8% (24/43)
Injection site pain	46.4% (26/56)	20.9% (9/43)
Headache	30.3% (17/56)	2.3% (1/43)
Chills	25.0% (14/56)	7.0% (3/43)
Fever	23.2% (13/56)	2.3% (1/43)
Arthralgia	21.4% (12/56)	4.7% (2/43)
Fatigue	17.9% (10/56)	20.9% (9/43)
Myalgia	16.1% (9/56)	–
Eye pain	10.7% (6/56)	–
Weakness	8.9% (5/56)	–
Paresthesia	1.8% (1/56)	2.3% (1/43)
Symptom duration, hr		
12–24	46.1% (18/39)	68.7% (11/16)
36–48	35.9% (14/39)	18.7% (3/16)
> 48	12.8% (5/39)	12.5% (2/16)
Medication		
None	48.2% (27/56)	60.0% (24/40)
Acetaminophen	50.0% (28/56)	37.5% (15/40)
Aspirin	–	2.5% (1/40)
Panadeine F	1.8% (1/56)	–

Our study is the first assessment of the antibody response to AZD1222 vaccination in the Caribbean region, and it provides important information related to common side effects experienced. Most importantly, we show that almost all (70 of 71) AZD1222 vaccine recipients in our study developed SARS-CoV-2 RBD IgG levels that remained positive 16 to 22 weeks after the second AZD1222 dose.

We observed a lower RBD IgG response to AZD1222 than a previous study in the UK that showed a median SARS-CoV-2 RBD IgG of 435 AU/mL for samples collected more than 21 days after the first AZD1222 dose using the same Abbott SARS-CoV-2 IgG II Quant assay.^8^ In our study, we showed a more modest median response of 303.5 AU/mL 3 to 7 weeks after the first AZD1222 dose, which could possibly be explained by a younger age of the UK population examined and/or genetic or other differences between populations.

The average time to the second AZD1222 dose in our study was 9 weeks. The duration between doses can affect the antibody response, with previous studies showing that receipt of the second AZD1222 dose at 12 weeks resulted in greater antibody levels compared with boosting at 8 weeks.^15^ More recent data show that extended periods between the first and second doses greater than 12 weeks is associated with higher antibody titers.^16^ Although extended times from initial dosing to the second dose may be beneficial, consideration must be given to the extent of SARS-CoV-2 circulation within populations, which may favor shorter intervals between doses.

Our study was limited by its modest sample size and neutralizing antibody testing that was not done. Although the SARS-CoV-2 IgG II Quant assay measures IgG antibodies against the spike RBD, a key domain associated with neutralization, the assay does not measure neutralizing antibodies directly. However, RBD IgG levels as measured with the Abbott SARS-CoV-2 IgG II Quant assay were shown previously to be correlated with neutralizing antibodies,^17^ and recent studies show that RBD IgG antibody levels are associated with vaccine efficacy.^18^^,^^19^ These studies examining correlates of protection indicate the potential utility of measuring RBD IgG antibody levels in the broader population. However, a correlate of protection is not universally agreed upon and may change with the emergence of new variants. Future assessment of a larger, nationally representative Jamaican population with assessment of neutralizing antibodies would provide more generalizable information, and should be considered by public health officials and taken in context with circulating variants.

In conclusion, our study shows that AZD1222 vaccination is associated with mild side effects in the Jamaican population and almost always results in RBD IgG levels that are sustained for at least 22 weeks after vaccination, providing evidence-based support for the continued use of AZD1222 in the English-speaking Caribbean.

## Supplemental Material


Supplemental materials
