# Supplementary material for: SARS-CoV-2 Receptor-Binding Domain IgG Response to AstraZeneca (AZD1222) COVID-19 Vaccination, Jamaica
Source: Am J Trop Med Hyg. 2022 Mar 23;106(5):1511–4. doi: 10.4269/ajtmh.21-1128 (PMC9128703; doi:10.4269/ajtmh.21-1128)
Supplement: Supplementary file 1 [file tpmd211128.SD1.pdf]

Supplemental Table 1

Spike RBD IgG response to first AZD1222 dose in persons with evidence of previous SARS-CoV-2 infection

Study ID	Days After First AZD1222 Dose	Spike RBD IgG, AU/mL	Nucleocapsid IgG, S/CO	Spike IgM, S/CO
7	28	511.8	1.05	0.44
20[#]	9	11,724.5	7.43	0.58
25[#]	29	18,314.3	8.11	9.64
29[#]	35	15,178.5	*	*
41	35	49.8	0.57	0.1
45[@]	21	6,178.1	3.33	1.44

[#]SARS-CoV-2 PCR-confirmed infections. PCR confirmation was 36 days, 26 days and 52 days before first AZD dose for study ID number 20, 25, and 29, respectively. [@]SARS-CoV-2 antibody positive 151 days prior to sampling. [*]Insufficient volume for testing. Study ID 20, 25, 29, and 45 reported COVID-19 symptoms. COVID-19 symptoms were not reported for study ID 7 and 41.

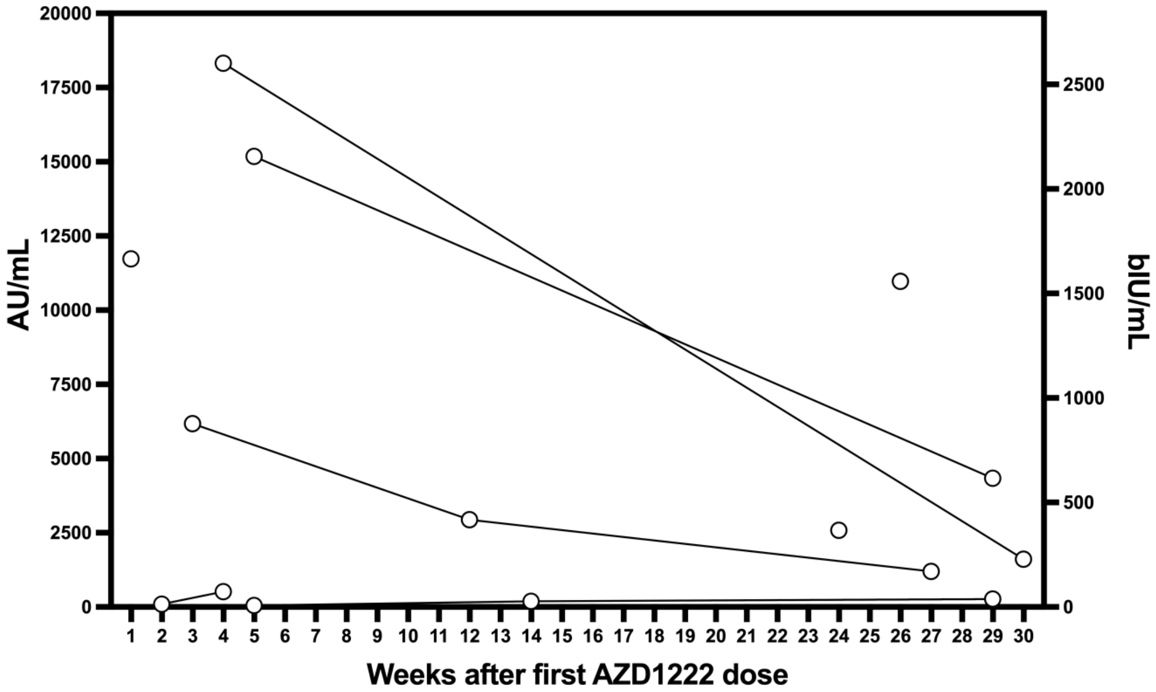

Supplemental Figure S1. SARS-CoV-2 RBD IgG antibody levels after AZD1222 vaccination for persons with previous evidence of SARS-CoV-2 infection prior to vaccination. SARS-CoV-2 RBD IgG was measured with the Abbott ARCHITECT SARS-CoV-2 IgG II Quant assay with results in arbitrary units per milliliter (AU/mL) on the left y-axis and WHO binding international units per milliliter (bIU/mL) on the right y-axis.

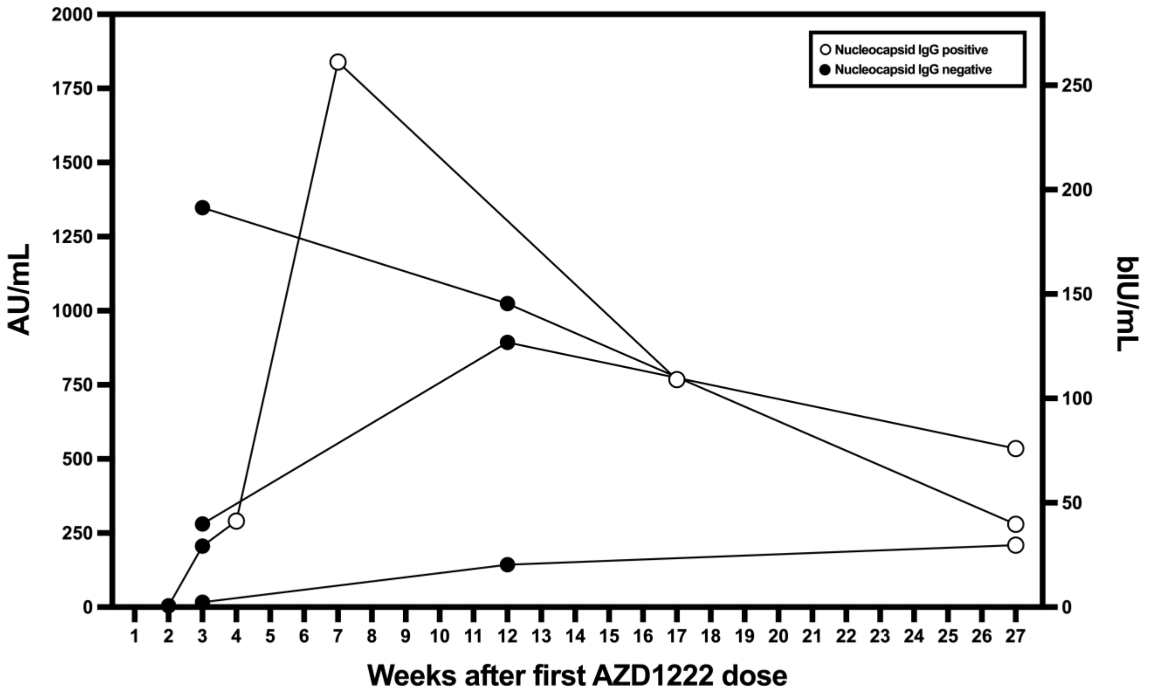

Supplemental Figure S2. SARS-CoV-2 RBD IgG antibody levels after AZD1222 vaccination for persons with evidence of SARS-CoV-2 infection after vaccination. SARS-CoV-2 RBD IgG was measured with the Abbott ARCHITECT SARS-CoV-2 IgG II Quant assay with results in arbitrary units per milliliter (AU/mL) on the left y-axis and WHO binding international units per milliliter (bIU/mL) on the right y-axis. COVID-19 symptoms were not reported for all persons.

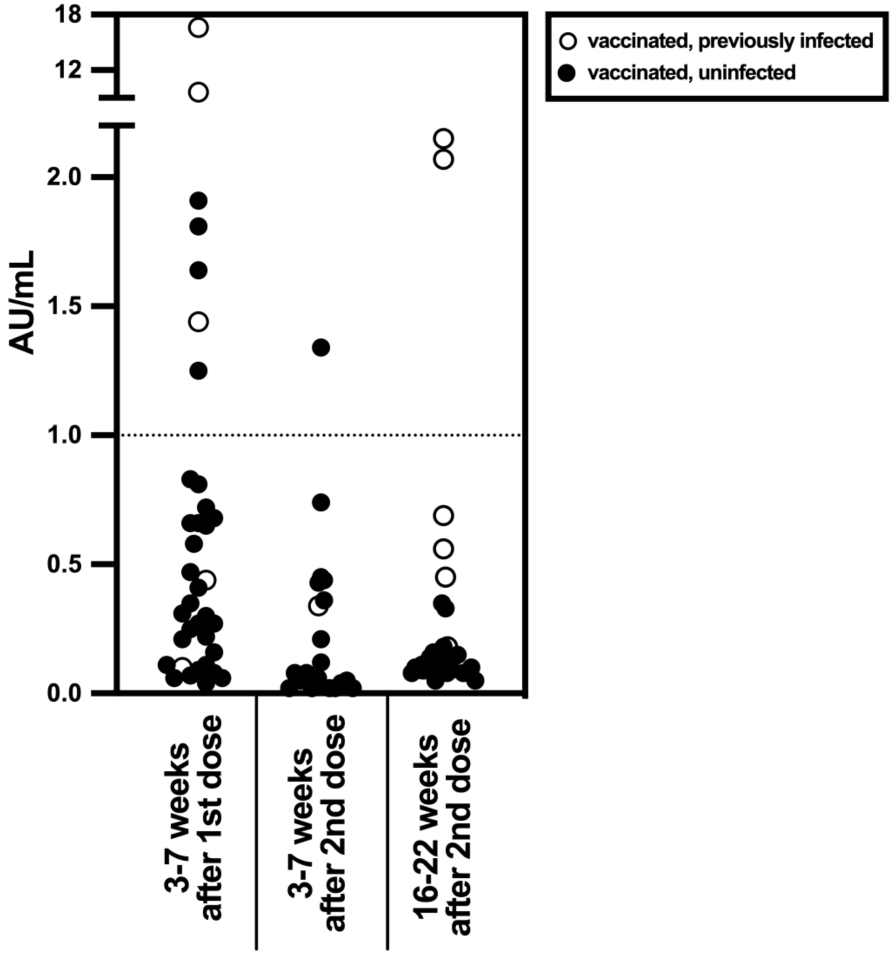

Supplemental Figure S3. SARS-CoV-2 spike IgM antibody levels after AZD1222 vaccination for persons with and without evidence of previous SARS-CoV-2 infection. SARS-CoV-2 spike IgM was measured with the Abbott ARCHITECT SARS-CoV-2 IgM assay with results in arbitrary units per milliliter (AU/mL). Sera was collected from AZD1222 vaccinated persons 3-7 weeks after first and second dose, and 16-22 weeks after second dose.
